# The effects of performance-based financing on neonatal health outcomes in Burundi, Lesotho, Senegal, Zambia and Zimbabwe

**DOI:** 10.1093/heapol/czaa191

**Published:** 2021-01-25

**Authors:** Anna Gage, Sebastian Bauhoff

**Affiliations:** Department of Global Health and Population, Harvard T.H. Chan School of Public Health, 665 Huntington Avenue, Building 1, 11th Floor, Boston, MA 02115, USA; Department of Global Health and Population, Harvard T.H. Chan School of Public Health, 665 Huntington Avenue, Building 1, 11th Floor, Boston, MA 02115, USA

**Keywords:** Health financing, health systems, health facilities, maternal and child health, quality of care, health care utilization

## Abstract

Maternal and newborn care has been a primary focus of performance-based financing (PBF) projects, which have been piloted or implemented in 21 countries in sub-Saharan Africa since 2007. Several evaluations of PBF have demonstrated improvements to facility delivery or quality of care. However, no studies have measured the impact of PBF programmes directly on neonatal health outcomes in Africa, nor compared PBF programmes against another. We assess the impact of PBF on early neonatal health outcomes and associated health care utilization and quality in Burundi, Lesotho, Senegal, Zambia and Zimbabwe. We pooled Demographic and Health Surveys and Multiple Indicator Cluster Surveys and apply difference-in-differences analysis to estimate the effect of PBF projects supported by the World Bank on early neonatal mortality and low birthweight. We also assessed the effect of PBF on intermediate outputs that are frequently explicitly incentivized in PBF projects, including facility delivery and antenatal care utilization and quality, and caesarean section. Finally, we examined the impact among births to poor or high-risk women. We found no statistically significant impact of PBF on neonatal health outcomes, health care utilization or quality in a pooled sample. PBF was also not associated with better health outcomes in each country individually, though in some countries and among poor women PBF improved facility delivery, antenatal care utilization or antenatal care quality. There was no improvement on the health outcomes among poor or high-risk women in the five countries. PBF had no impact on early neonatal health outcomes in the five African countries studied and had limited and variable effects on the utilization and quality of neonatal health care. These findings suggest that there is a need for both a deeper assessment of PBF and for other strategies to make meaningful improvements to neonatal health outcomes.

**KEY MESSAGES**

- We estimated the impact of performance-based financing (PBF) in Burundi, Lesotho, Senegal, Zambia and Zimbabwe on early neonatal death and low birthweight using a difference-in-differences approach.
- PBF did not reduce early neonatal mortality or low birthweight in across the five countries or in any country individually. There was also no improvement on the health outcomes among poor or high-risk women.
- There is a need for a deeper assessment of the costs and benefits of PBF projects and development of other strategies to improve neonatal health outcomes.

## Introduction

Despite decades of declining neonatal mortality rates, many countries in sub-Saharan Africa are still not on track to reach the Sustainable Development Goal of 12 neonatal deaths per 1000 live births by 2030. Maternal and newborn care services provided in health care facilities are viewed as critical to accelerate progress on neonatal health outcomes (Gülmezoglu *et al.*, 2016; World Health Organization, 2019).

Improving the quantity and quality of maternal and newborn care services has been a primary focus of performance-based financing (PBF) projects in Africa in the past two decades, partly driven by support from the Health Results Innovation Trust Fund (HRITF) administered by the World Bank (Kandpal, 2016; Gergen *et al.*, 2017). While there are many models of PBF, these projects generally entail a set of financing reforms that explicitly incentivize pre-defined quantity and quality indicators (Renmans *et al.*, 2017). Through incentives, PBF aims to motivate providers to improve their performance, help attract more capable health workers, or provide additional funding that can support improvements (Lemière *et al.*, 2013). Commonly incentivized maternal and newborn service indicators include the volume of antenatal care visits and facility deliveries and quality measures, such as all deliveries being conducted by qualified personnel and presence of proper maternity equipment (Gergen *et al.*, 2017).

A robust literature documents the variable impacts of African PBF projects on the quantity and quality of health care services. An influential early evaluation found that Rwanda’s PBF raised the number of facility deliveries and the quality of antenatal care, among other intermediate outputs, but did not improve the number of antenatal care visits (Basinga *et al.*, 2011). Recent reviews similarly found that incentivizing health facilities to provide deliveries can increase their number, but mixed evidence on quality of care and quantity of antenatal care with variation across projects and indicators within projects (Witter et al., 2012; Lemière *et al.*, 2013; Kandpal, 2016).

There is currently no empirical evidence on the direct impact of PBF on neonatal health outcomes in African countries. Several studies have modelled health impacts of PBF based on changes to utilization and quality (Zeng et al., 2018; Chinkhumba *et al.*, 2020), but direct evidence is critical for several reasons. First, changes in intermediate outputs may not always translate to better health. For example, increasing facility delivery may not improve neonatal health outcomes in the absence of high-quality care (Lim *et al.*, 2010; Fink *et al.*, 2015), and improved adherence to evidence-based checklists during delivery can fail to generate better maternal or newborn health outcomes (Semrau *et al.*, 2015). Second, the evaluations to date have demonstrated mixed results, with improvements on some indicators, generally including facility delivery, but not on others, including delivery quality (Eichler *et al.*, 2013; Turcotte-Tremblay *et al.*, 2016). It is unclear how these inconsistent improvements may come together to affect health outcomes, and the modelling studies rely on strong assumptions about quality-adjusted coverage measures (Zeng *et al.*, 2018; Chinkhumba *et al.*, 2020). Third, PBF projects incentivize a particular set of indicators and it remains largely unclear whether there are negative or positive spill-overs. For example, PBF may inadvertently divert resources and attention but could also encourage closely associated beneficial behaviours that are not incentivized (Lemière *et al.*, 2013; Sherry, 2016; Sherry *et al.*, 2017). Finally, PBF projects generally pursue multiple strategies, so that focussing on intermediate outputs may miss other pathways to improved health outcomes. Examining the direct impact on health outcomes captures all pathways and spill-overs that are otherwise difficult to model in the context of complex adaptive systems (Paina and Peters, 2012). As improving maternal and child health outcomes, including neonatal health outcomes, is a primary objective of many PBF projects, it is important to evaluate these impacts directly (Bonfrer *et al.*, 2014; Friedman *et al.*, 2016a,b).

In this paper, we empirically evaluate the impact of five PBF projects in Africa on two important neonatal health outcomes, neonatal mortality and low birthweight, as well as on intermediate outputs through which PBF may improve health outcomes: antenatal care utilization and quality, facility delivery utilization and quality, and caesarean section rates. We conduct both pooled and country-specific analyses, and also assess the impact of PBF for two vulnerable groups: poor women and women with high-risk births.

Our analysis offers three primary contributions. First, we provide direct evidence of the impact of African PBF projects on neonatal mortality, avoiding the challenges faced by modelling studies. Second, we compare the effectiveness of PBF projects in different countries against one another using the same methods and data. Most evaluations focus on just one project and because they use differing methodologies, they are not directly comparable (Oxman and Fretheim, 2009; Eichler *et al.*, 2013). As each project is implemented differently, a direct comparison can help to identify features of the health system context or project that may be more or less effective. Finally, our analysis represents a systematic replication of previous evaluations using alternative data sources (Bonfrer *et al.*, 2014).

## Materials and methods

### Data and study countries

Our analysis focussed on PBF projects in five African countries: Burundi, Lesotho, Senegal, Zambia and Zimbabwe. Countries were included into the study if they were in sub-Saharan Africa, had implemented an PBF project supported by the World Bank’s HRITF and for which the intervention provinces or districts are known, and had a publicly available nationally representative survey on health care and utilization both prior to and after implementation of the PBF project. Although Burundi did not have a survey prior to its PBF implementation, we were able to include Burundi by using just the post-DHS survey for a longer span of births. The DHS collects data on neonatal mortality for all births of the women respondents regardless of when the birth occurred. Burundi is excluded from the pooled analysis as a robustness check. Countries that assigned PBF to specific facilities or sub-districts within districts were further excluded from the study, as in this study the population’s treatment status was assigned by their district residence rather than by facility catchment areas.

We used the Demographic and Health Surveys (DHS) and Multiple Indicator Cluster Surveys (MICS) to assess the impact of PBF. Because there were differing amounts of time between the surveys and the PBF implementation in each country, we limited the analysis to births that occurred within 3 years before implementation and 2 years after. We also excluded all births from mothers outside of the defined treatment and control districts. In Zambia and Zimbabwe, data on the household’s district were not available directly from the surveys. In these cases, we used the cluster geocodes to place households in districts. Although DHS geocodes are displaced to maintain privacy, the displacement is restricted so that clusters stay within the second administrative level, or the district, in these countries (Burgert *et al.*, 2013).

We assumed that a household was treated if it was located within a PBF implementation district, and therefore that all facilities within implementation districts were treated and that women would have gone to facilities within her district. Table 1 summarizes the data sources used for each country.

**Table 1:** PBF characteristics and data sources

	Burundi	Lesotho	Senegal	Zambia	Zimbabwe
First implementation date	December 2006	July 2016	April 2012	April 2012	March 2012
Second implementation date	October 2008	October 2016	N/A	N/A	N/A
Additional rollout	Expanded to control regions in April 2010	N/A	Expanded to control regions in May 2016	Expanded to 39 districts in October 2016	Expanded to 44 districts in 2015
Pre-implementation survey	DHS 2010^a^	DHS 2014	DHS 2011	DHS 2007	DHS 2010 − 11
Post-implementation survey	DHS 2010 and DHS 2017	MICS 2018	Continuous DHS 2013 − 17	DHS 2014 and DHS 2018	DHS 2015
District selection notes				Randomized treatment to districts. Additional unconditional financing arm in 10 districts.	Government selected implementation districts from pair-matched districts.
Major related concurrent interventions		Incentives for district teams for good quality of supervision and support to PBF project	Demand-side vouchers also provided for four ANC visits and skilled delivery	.	Introduced simultaneously with national elimination of user fees for targeted services.
Payment adjustment on other dimensions	Remoteness, poverty, staff and facility needs	Remoteness		Remoteness	Remoteness
Allocation of PBF payment					
Health facility	70%	50%	25%	40%	75%
Staff incentives	30%	50%	75%	60%	25%

a Given the absence of earlier data sources in Burundi, we used the birth recode file from 2010 for the pre-implementation survey through including births that occurred prior to implementation.

### PBF projects

The PBF projects differed in their design and implementation across the study countries. In general, the projects were structured to provide healthcare facilities financial incentives conditional on reaching certain performance targets. Maternal and newborn care was a priority for all of the study countries, and targets included both quantity and quality of services. The volume of facility deliveries provided by a skilled birth attendant and antenatal care visits were rewarded in all study countries. Quality measures included structural quality items, such as water and soap available in delivery room (Lesotho), and process quality measures, such as correct use of the partograph (Senegal). The programs all had quantity-based formulas for determining the incentive, which were then inflated (or deflated, in Senegal) based on a quality score. None of the projects directly rewarded improvements on early neonatal death or low birthweight. Further details about the implementation and incentivized measures are provided in Supplementary Appendix 1.

Four of the five study countries employed purposive selection to select the districts for PBF implementation. For example, in Zimbabwe districts were pair-matched on baseline characteristics such as geographic accessibility and average catchment population and then government officials selected between the two districts for implementation. Implementation was randomized only in Zambia, where districts were also matched prior to randomization. In addition, Zambia also had a third treatment arm which gave facilities unconditional financing equivalent to the amount of the PBF arm. We use the pure control districts without unconditional financing as the controls in the primary analysis but conduct a sensitivity analysis which compares the conditional and unconditional arms in Zambia.

We selected control districts in Zambia and Zimbabwe to match those from the World Bank’s impact evaluations (Friedman *et al.*, 2016a,b). Burundi and Senegal both implemented a phased rollout; consequently, we defined the control districts as those that later received PBF in those countries (Bonfrer *et al.*, 2014; Falisse *et al.*, 2015). The additional rollouts did not occur within the time period considered in this study. Finally given the small size of Lesotho, we defined the control districts as all the remaining districts that had not received PBF. We excluded Quthing and Leribe districts in Lesotho because they had piloted PBF 2 years prior to the larger implementation of PBF (The World Bank, 2017). Supplementary Appendix 1 lists of all the implementation and control districts for each country. In a sensitivity analysis, we use all non-implementation districts in all of the countries as controls, only excluding districts that had a prior pilot implementation.

Burundi and Lesotho rolled out the PBF project in two stages within the study period. In the primary analysis, we consider only the first set of implementation districts and the control districts; in a sensitivity analysis we separately compare the second set of implementation districts against the control districts.

### Dependent variables

We examined the effect of the PBF projects on two primary neonatal health outcomes: early neonatal death and low birthweight. Early neonatal death, which is associated with facility delivery and quality (Fink *et al.*, 2015; Leslie *et al.*, 2016), was defined as a death before or including 7 days of birth. Low birthweight, which is associated with ANC quantity and content (Coria-Soto *et al.*, 1996), was defined as a birthweight below or including 2500 g. If the baby was not weighed at birth, we used multiple imputation with five imputations to impute missing values based on the mother’s report of the baby’s size at birth and risk factors including multiple births, primipara, urban location, maternal age and primary education, wealth quintile and district (Katz *et al.*, 2013). Although there may be measurement error in the mother’s report of the baby’s size, this measure is strongly correlated with related health outcomes such as prematurity and intrauterine growth restrictions (McClure *et al.*, 2011; Fink *et al.*, 2015). As robustness checks, we also tested whether PBF impacted the likelihood of birthweight being recorded and the impact of PBF on the subset of observations where birthweight was recorded.

We also examined several pathways through which PBF might affect these health outcomes, including increased utilization or improved quality of antenatal or intrapartum care or increased caesarean sections. We defined antenatal utilization as at least four antenatal care visits and intrapartum utilization as delivering in a health facility. Antenatal and delivery quality were both defined as binary variables, where high quality care recipients received all of the recommended quality items while low-quality care recipients received fewer items. Quality measures were alternatively defined as the percent of items received as a robustness check. Antenatal care quality items included the recommended number of Tetanus Toxoid shots, iron supplementation, a blood sample test and antenatal care from a qualified provider. Iron supplementation was not measured in the 2018 Lesotho MICS, so quality in Lesotho during both waves was measured using the other three items. Delivery quality items included breastfeeding within an hour of delivery, postnatal check before discharge and delivery with a trained provider. Finally, caesarean section was defined as the mother’s report of a caesarean delivery.

The sample for each dependent variable varied based on data availability. Neonatal death data was available for all births, and we imputed birthweight for all births as described above. Antenatal care utilization and quality were only collected for the most recent birth; delivery quality was collected about the most recent birth if the woman had a facility delivery. Facility delivery and caesarean sections were collected about all births.

### Analysis

We pooled data from all study countries and used a difference in differences specification to assess the impact of PBF on the study dependent variables.

\begin{equation*} {Y_{{\text{idt}}}} = {\beta _0} + {\beta _1}\left( {{\text{PB}}{{\text{F}}_d}{\text{*Pos}}{{\text{t}}_t}} \right) + {\beta _2}{\gamma _{idt}} + \mathop \sum \limits_{j = 1}^{60} {\beta _j}{\text{*Mont}}{{\text{h}}_t} + \mathop \sum \limits_{k = 1}^{75} {\beta _k}{\text{*Distric}}{{\text{t}}_d} + {\varepsilon _{idt}}, \end{equation*}

where *Y* is a dependent variable for an individual *i* in district *d* and month *t*, PBF is an indicator for whether the district was treated, Post is an indicator for whether the birth was after the date of implementation, *Y* is a set of covariates, Month is a set of fixed effects of the month of birth in relation to the date of implementation where PBF was implemented in month 37, and District is a set of district fixed effects. We used multivariable linear probability models with standard errors clustered by district. We similarly tested for parallel pre-trends between implementation and control districts by interacting quarter fixed effects prior to and after the PBF implementation with the binary PBF indicator, excluding the quarter that PBF was implemented. This method can also be used to examine the effect of PBF over time.

Because PBF was not randomized to districts in most countries, we both matched on a set of covariates and controlled for them in our model to obtain a better balance on important characteristics and improve the precision of our estimates (Chen *et al.*, 2016). We used coarsened exact matching (CEM) to first match births on the set of covariates. CEM is a method that corrects for imbalances between composition of treatment and control districts by coarsening a set of covariates into bins, creating a stratum per bin and assigning observations to the strata, then dropping any births whose stratum does not contain at least one treated and one control unit (Blackwell *et al.*, 2009; Chen *et al.*, 2016). We included covariates that are known to be associated with neonatal health outcomes, including multiple birth, primipara, maternal age, year of birth, mother’s completion of primary education, urban vs rural location, and whether the household is in the poorest two wealth quintiles in the country. We included these covariates directly in the model in addition to using the CEM weights in order to further control potential residual confounding and improve precision (Blackwell *et al.*, 2009).

We conducted several additional analyses to understand whether the effect differed among sub-populations of interest. First, we conducted the differences in differences model separately in each study country in addition to the pooled analysis. We did not further adjust the standard errors for the small number of clusters in some countries; doing so would result in even more conservative results. Second, we ran the pooled model among the subset of households that were in the poorest two wealth quintiles in the country and among the subset of high-risk births. We defined high-risk births as those to primipara women, to women younger than 18 years or older than 34, or multiple births.

Descriptive statistics are presented with the DHS and MICS sampling weights. Analyses were conducted in Stata 15. The original survey implementers obtained ethical approvals for data collection; the authors’ institute approved this secondary analysis as exempt from human subjects review.

## Results

A total of 30 200 births from DHS or MICS across the five study countries met the inclusion criteria for the study. These included 12 790 births born after the introduction of PBF in their respective countries and 12 700 births that occurred in districts that implemented PBF projects. After CEM, 28 619 births were retained in the analysis, removing 1016 births from control districts and 565 births from PBF districts that were not matched.

Table 2 displays the study outcomes and key covariates by treatment district prior to PBF implementation among the matched sample. Across the study countries, 658 (2.3%) births resulted in early neonatal death, ranging from 174 (1.5%) in Senegal to 99 (3.5%) in Lesotho. A total of 4579 (16%) births were low birthweight. Facility delivery and antenatal care utilization rates were low in most countries prior to the intervention; only Lesotho had over 70% facility delivery rate and only 55% of births had four antenatal care visits. Birthweight was recorded on a card for less than half of births at baseline; the PBF interventions did not have an impact on whether birthweight was recorded (Supplementary Appendix 4).

**Table 2:** Dependent variables and covariates in control and implementation districts prior to implementation among analytic sample

	Burundi		Lesotho		Senegal		Zambia		Zimbabwe		Total	
	Control	PBF	Control	PBF	Control	PBF	Control	PBF	Control	PBF	Control	PBF
Districts	6	3	4	4	4	2	10	10	16	16	40	35
Pre-implementation births	3229	1557	1013	980	3576	2499	931	990	729	993	9478	7019
Post-implementation births	2285	1217	427	418	3300	2244	602	611	392	626	7006	5116
Pre-implementation dependent variables												
Early neonatal death	2.3%	3.2%	3.7%	3.2%	1.3%	1.7%	1.5%	1.6%	2.4%	3.0%	2.2%	2.5%
Low birthweight	18%	20%	13%	16%	21%	17%	15%	14%	13%	15%	17%	16%
Facility delivery	49%	50%	84%	76%	57%	46%	54%	59%	74%	64%	61%	57%
Delivery quality	86%	86%	56%	58%	55%	53%	71%	72%	54%	58%	61%	61%
C-section	1%	3%	13%	8%	3%	1%	2%	3%	5%	4%	4%	3%
4+ ANC visits	31%	38%	75%	69%	43%	32%	60%	59%	69%	63%	57%	52%
ANC quality	8%	0%	61%	60%	55%	49%	45%	44%	30%	30%	48%	43%
Pre-implementation covariates												
Mother’s age at birth (mean)	26.7	27.0	25.2	25.5	26.6	26.2	26.3	26.2	25.9	25.5	26.3	26.2
Mother primary education	38%	45%	100%	100%	31%	18%	88%	90%	99%	99%	57%	62%
Primipara	20%	23%	44%	39%	20%	19%	20%	17%	27%	29%	24%	24%
Multiple birth	0%	1%	0%	1%	1%	2%	2%	1%	1%	2%	1%	1%
Urban	2%	3%	46%	19%	24%	13%	16%	11%	27%	23%	17%	13%
Poorest wealth quintile	21%	22%	13%	37%	41%	59%	28%	38%	28%	38%	25%	37%
Birthweight recorded	7%	8%	44%	46%	45%	30%	51%	61%	51%	61%	32%	39%

Treatment and control districts were not balanced on all covariates prior to PBF implementation even after matching. PBF was implemented more often in poorer districts, particularly in Lesotho, Senegal and Zimbabwe, and in rural districts. Despite these differing characteristics, the trends in most outcomes do not significantly differ between implementation and control districts prior to implementation (Supplementary Appendix 3).

**Figure 1:**
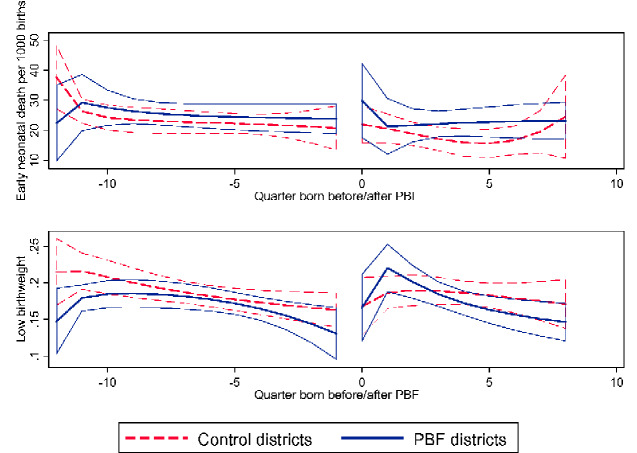
Pooled unadjusted trends in early neonatal death and low birthweight before and after PBF implementation.

Table 3 presents the results from the difference in differences estimation pooling together births from all the study countries and stratified by country. We found no statistically or substantially significant effect of the PBF intervention on any of the health outcomes or intermediate outputs in the pooled analysis. The unadjusted trends for early neonatal death and low birthweight are shown in Figure 1, while the results for the intermediate outputs are shown in Supplementary Appendix 2. These results were robust to excluding Burundi, to using all non-implementation districts as controls, to using the alternative definitions of the quality measures, to only including observations where birthweight was recorded, and to using the second implementation date in Burundi and Lesotho (Supplementary Appendix 4). There also do not appear to be delayed effects of PBF within the 2-year period assessed (Supplementary Appendix 3).

**Table 3:** Effects of PBF on primary and secondary outcomes pooled and in all study countries

	Pooled	Burundi	Lesotho	Senegal	Zambia	Zimbabwe
Early-neonatal death						
Coef.	0.00	0.00	0.02	0.00	−0.01	0.00
95% CI	(−0.01, 0.01)	(−0.01, 0.01)	(−0.01, 0.05)	(−0.01, 0.01)	(−0.04, 0.01)	(−0.02, 0.03)
*N*	28 619	8288	2838	11 619	3134	2740
Low birthweight						
Coef.	0.01	0.01	−0.05	0.03	0.00	−0.02
95% CI	(−0.02, 0.03)	(−0.12, 0.13)	(−0.14, 0.03)	(−0.01, 0.08)	(−0.06, 0.06)	(−0.1, 0.06)
*N*	28 619	8288	2838	11 619	3134	2740
Facility delivery						
Coef.	0.03	**0.08**	0.03	0.03	0.03	−0.02
95% CI	(−0.01, 0.07)	**(0.02, 0.14)**	(−0.04, 0.09)	(−0.03, 0.08)	(−0.06, 0.12)	(−0.1, 0.06)
*N*	21 471	**2140**	1849	11 619	3123	2740
Delivery quality						
Coef.	−0.05	−0.05	−0.09	−0.05	−0.05	−0.03
95% CI	(−0.14, 0.04)	(−0.16, 0.06)	(−0.24, 0.06)	(−0.11, 0.01)	(−0.16, 0.06)	(−0.14, 0.09)
*N*	13 054	1219	1558	6275	2026	1976
C-section						
Coef.	0.00	0.01	−0.01	0.00	0.00	−0.02
95% CI	(−0.01, 0.01)	(−0.03, 0.05)	(−0.11, 0.09)	(−0.01, 0.01)	(−0.03, 0.04)	(−0.05, 0.01)
*N*	21 424	2145	1849	11 564	3128	2738
ANC 4 visits						
Coef.	0.04	−0.06	**0.12**	0.02	0.06	0.00
95% CI	(−0.02, 0.10)	(−0.18, 0.07)	**(0.01, 0.22)**	(−0.1, 0.15)	(−0.02, 0.13)	(−0.12, 0.13)
*N*	14 383	793	**1840**	7383	2157	2210
ANC quality						
Coef.	0.02	0.09	−0.03	0.03	**0.09**	−0.03
95% CI	(−0.04, 0.09)	(−0.05, 0.24)	(−0.14, 0.08)	(−0.1, 0.16)	**(0.01, 0.17)**	(−0.16, 0.09)
*N*	14 510	796	1869	7445	**2172**	2228

Bolded estimates signify confidence intervals that do not contain zero. Estimated coefficients for β^1^ from multivariable difference-in-difference regressions representing the percentage point change in the outcome, with standard errors clustered at the district level.

Consistent with the pooled results, PBF did not have a significant effect on early neonatal death or low birthweight in any of the study countries. Zambia’s PBF may have resulted in a slight decline in early neonatal death, but the 95% confidence interval (CI) contained zero. However, several countries did see some effect on intermediate outputs. Facility delivery rose 8 percentage points in Burundi (95% CI: 0.02, 0.14), antenatal care visits rose by 12 percentage points in Lesotho (95% CI: 0.01, 0.22) and antenatal visit quality improved by 9 percentage points in Zambia (95% CI: 0.01, 0.17). There were no effects on delivery quality or caesarean sections in any country. In Zambia, there were no effects on the primary or secondary outcomes when comparing the PBF districts to the unconditional financing arm rather than the pure control arm (Supplementary Appendix 4).

Table 4 presents the results when the pooled sample is restricted to the two sub-populations of interest. PBF increased antenatal care utilization by 8 percentage points (95% CI: 0.00, 0.17) among poor women. It did not have any effect on the health outcomes or any of the other intermediate outputs in either of the populations of interest.

**Table 4:** Effects of PBF on primary and secondary outcomes among populations of interest

		Poor women			High-risk births	
Outcome	Coef.	95% CI	*N*	Coef.	95% CI	*N*
Early neonatal death	0.00	(−0.01, 0.02)	9680	0.00	(−0.01, 0.02)	10 887
Low birthweight	0.00	(−0.03, 0.03)	9680	0.01	(−0.04, 0.05)	10 887
Facility delivery	0.02	(−0.04, 0.09)	8051	0.03	(−0.02, 0.09)	8222
Delivery quality	−0.05	(−0.16, 0.06)	3476	−0.07	(−0.18, 0.05)	5570
C-section	−0.01	(−0.02, 0.01)	8034	−0.01	(−0.03, 0.01)	8205
ANC 4 visits	**0.08**	**(0, 0.17)**	5122	0.04	(−0.02, 0.1)	5771
ANC quality	0.06	(−0.03, 0.14)	5152	0.00	(−0.06, 0.07)	5824

Bolded estimates signify confidence intervals that do not contain zero. Estimated coefficients for β^1^from pooled multivariable difference-in-difference regression, with standard errors clustered at the district level.

## Discussion

PBF is considered an innovative approach to tackle the challenges to improving neonatal health outcomes that persist in many African countries. This study used quasi-experimental methods and population representative secondary data to assess the effect of PBF projects on neonatal health outcomes, and the quantity and quality of care in five African countries. Despite the large sample sizes from pooling the data, we found no effect on any of the examined outputs or outcomes. Although there were several positive impacts on utilization and antenatal care quality among individual country projects and among poor women, no project had a statistically detectable impact on either neonatal mortality or low birthweight. Furthermore, the PBF projects did not have detectable impacts on the health outcomes for two vulnerable sub-groups, poor women and women with a high-risk birth.

There may be several reasons for our null findings. First, the potential of PBF may be constrained by the ability of health facilities or providers to adjust their behaviour to improve performance. In practice, they may already be operating at capacity given their environmental, educational and structural constraints. For example, chronic staff shortages limited sustained improvement in Zimbabwe (Moyo *et al.*, 2015). Poorly functioning health systems may instead require greater foundational change than adjustments to provider performance (Kruk *et al.*, 2018). Second, PBF may have both positive and negative effects on different aspects of provider motivation (Shen *et al.*, 2017; Lohmann *et al.*, 2018), and its effects on non-incentivized services can be ambiguous (Sherry, 2016; Sherry *et al.*, 2017). Although improving health outcomes is a stated primary goal of all PBF projects in this study, it is possible that the projects had positive impacts on important clinical and non-clinical areas that we did not consider. Third, the specific design and implementation of the projects could affect their impacts. For example, the incentives may be too low or not be tied to the most effective behaviours. This may be particularly relevant for quality of care: PBF predominantly incentivizes structural quality (Gergen *et al.*, 2017), which may be only weakly correlated with care processes (Leslie *et al.*, 2017).

Despite the large-pooled sample size, the study may also still not be adequately powered to detect changes in early neonatal death. An ex-post power calculation (Supplementary Appendix 5) suggests that the minimum detectable effect is a 0.67 percentage point change in the probability of early neonatal death, with the available sample size, 80% power and a 5% significance level. Smaller changes may be policy relevant, however, the small coefficient size and lack of effect in any of the intermediate outputs suggests that an effect would still not be detectable even with a larger sample size.

Some of our results differ from those of earlier impact evaluations of these PBF projects, which are summarized in Table 5. While no prior study had directly assessed the impacts on health outcomes, several studies found positive impacts on utilization or quality, particularly on rates of facility delivery (Bonfrer *et al.*, 2014; Friedman *et al.*, 2016a,b). We found a positive impact on facility delivery in Burundi, though smaller effect size than in earlier studies (Bonfrer *et al.*, 2014), and no impact in Zambia or Zimbabwe. There may be a number of explanations for this divergence, including differences in the sampling strategy, timing of data and inclusion criteria; differences in the covariates used to control for baseline differences; and our use matching to reduce covariate imbalance. There are also differences in how quality is measured. Our quality measures rely on a relatively small number of process measures from self-reports, whereas the earlier studies tend to use more indicators and rely more heavily on structural measures. For example, the Burundi evaluation uses a composite facility-based measure constructed using 57 structural and process indicators (Bonfrer *et al.*, 2014), while the large impact on delivery quality in Zambia is driven by the availability of equipment, medicines and supplies in the delivery room (Friedman *et al.*, 2016a,b).

**Table 5:** Summary of effects from previous impact evaluations

	Burundi^d^	Lesotho	Senegal	Zambia^e^	Zimbabwe^f^
Early neonatal death	Not assessed	Not assessed	Not assessed	Not assessed	Not assessed
Low birthweight	Not assessed	Not assessed	Not assessed	Not assessed	Not assessed
Facility delivery	22 pp	Not assessed	Not assessed	13 pp	13 pp
Delivery quality	17 pp^a^	Not assessed	Not assessed	57 pp	No effect
C-section	Not assessed	Not assessed	Not assessed	Not assessed	7 pp
ANC visits	No effect	Not assessed	Not assessed	No effect	No effect
ANC quality	17 pp^a^	Not assessed	Not assessed	Mixed^b^	Mixed^c^

Statistically significant effects reported; all reported effects were positive.

a Facility quality measured overall, rather than by service.

b Found improvements in iron supplementation and malaria drugs, decrease in urine sample taken, and no change in other 5 ANC quality measures assessed.

c Found improvements in urine sample taken and tetanus injections, and no change in other 6 ANC quality measures assessed.

d Bonfrer *et al.* (2014).

e Friedman *et al.* (2016b).

f Friedman *et al.* (2016a).

This study has a number of limitations. First, women’s treatment status may have been misclassified based on her district of residence at the time of the interview. This may be the case if the woman moved districts between the birth and the survey, sought care outside of her district, or visited a private facility which did not receive the RBF intervention within an RBF district. While these cases should affect a small per cent of women and should not differentially affect women in intervention or comparison districts, a misclassified status would bias the results towards the null. Second, the quality measures available in the DHS and MICS data sets were limited. We selected indicators for process quality that may have a large impact on neonatal health outcomes but only partially capture routine delivery and antenatal care quality. Third, the mostly non-randomized implementation of the PBF projects could result in residual confounding that persists despite matching at baseline. Although we found pre-trends to be largely parallel, there could be unobserved time-variant factors that differentially impacted the districts during the study period. Fourth, we were unable to look at a longer time frame beyond 2 years because of PBF implementation in the control areas in some of the countries at that time. Although neonatal mortality can be responsive to changes in the health system (Magge *et al.*, 2020), it may take longer than this period to see an effect particularly if there were delays in signing contracts or delivering payments (Rajkotia *et al.*, 2017; Ridde *et al.*, 2018). Finally, we were unable to look at treatment heterogeneity at levels lower than the country because of limited sample sizes.

The mixed and variable effects we observed across countries indicate scopes for learning from comparative studies. Such comparisons and innovations in measurement (e.g. of quality) can also be used to adjust ongoing projects (Fritsche and Peabody, 2018). The large number of HRITF-supported PBF pilots provides an important opportunity for such further research.

Overall, our results indicate that PBF—as implemented in the five projects we examined—may have limited impacts on neonatal health outcomes, as well as the associated utilization and quality pathways. While this does not preclude PBF from having other effects, positive or negative, this finding suggests caution with designing and deploying PBF with the goal of improving neonatal health outcomes at the population level. PBF may have other benefits, e.g. arising from increased autonomy and supervision (Renmans *et al.*, 2017), but must also contend with other criticisms, such the lack of domestic ownership and the diversion of attention and resources away from broader health systems strategies (Paul *et al.*, 2018; Ridde *et al.*, 2018). Different strategies will likely be needed to make meaningful progress on improving neonatal health outcomes in sub-Saharan Africa.

## Supplementary data

Supplementary data are available at *Health Policy and Planning* online.

## Funding

Publication fees were supported by The Bill and Melinda Gates Foundation grant number OPP1161450.

## Supplementary Material

czaa191_Supp
