# Supplementary material for: The effects of performance-based financing on neonatal health outcomes in Burundi, Lesotho, Senegal, Zambia and Zimbabwe
Source: Health Policy Plan. 2021 Jan 25;36(3):332–40. doi: 10.1093/heapol/czaa191 (PMC8058947; doi:10.1093/heapol/czaa191)
Supplement: czaa191_Supp [file czaa191_supp.zip › Appendix_RR_sb_ag.docx]

**Appendix 1. PBF implementation details**

The PBF projects differed in their design and implementation across the five study countries. This appendix provides more detailed information about the design and implementation in each country. First, we provide an overview of the types of indicators that were incentivized by the PBF programs. Second, we include descriptions of each project sourced from published documents.

*Incentivized indicators*

We examined all incentivized indicators in the PBF study countries from the TRAction database^1^ and categorized them into structural quality, process quality, or coverage indicators. Indicators were included if they were directly related to antenatal, labor and delivery, PMTCT or postnatal services, or if they were service agnostic but were applicable to maternal services (i.e. availability of electricity or handwashing). A total of 259 unique indicators were classified.

Structural quality indicators were further categorized into infection prevention items (i.e. sterile gloves); key equipment (i.e. baby weighing scale); medications (i.e. local anesthesia available); patient amenities (i.e. curtain between delivery bed and door); human resources (i.e. No absence of staff for unjustified reasons during last 3 months); record keeping (i.e. Admission card correctly filled out) and fees (i.e. delivery fee). Process quality was further categorized into distinct services: ANC (i.e. weight, blood pressure, breast exam and check for edema completed during physical examination); labor and delivery (i.e. APGAR noted during the 1^st^, 5^th^ and 10^th^ minutes); PMTCT (i.e. Proper monitoring of infants born to HIV positive mothers) and postnatal care (i.e. Postnatal consultations compliance with quality standards). Coverage indicators included indicators such as rate of women having postnatal consultation and number of uncomplicated deliveries.

Number of incentivized indicators by category

| Indicator type | Burundi | Lesotho | Senegal | Zambia | Zimbabwe |
| --- | --- | --- | --- | --- | --- |
| Structural quality |  |  |  |  |  |
| Infection prevention items | 8 | 15 | 3 | 0 | 0 |
| Key equipment | 34 | 18 | 5 | 12 | 7 |
| Medications | 5 | 8 | 0 | 2 | 1 |
| Patient amenities | 2 | 8 | 6 | 0 | 0 |
| Human resources | 3 | 3 | 5 | 0 | 0 |
| Record keeping | 4 | 13 | 4 | 1 | 0 |
| Fees | 1 | 1 | 0 | 0 | 4 |
| Process quality |  |  |  |  |  |
| ANC process | 2 | 14 | 3 | 14 | 3 |
| Delivery Process | 6 | 6 | 7 | 3 | 5 |
| PMTCT Process | 5 | 1 | 6 | 2 | 1 |
| Postnatal Process | 0 | 0 | 2 | 0 | 1 |
| Coverage | 13 | 14 | 5 | 9 | 11 |

The programs varied widely in the number and type of indicators. Zimbabwe’s program had 33 incentivized indicators relevant to maternity care while Lesotho’s had 101. Programs with more incentivized indicators tended to have a larger proportion in structural quality, while Zimbabwe’s were equally split between structural quality, process quality and coverage measures.

*Implementation and control districts*

|  | First implementation districts | Second implementation districts | Control districts | Unconditional financing districts |
| --- | --- | --- | --- | --- |
| Burundi | Bubanza, Cankuzo and Gitega | Makamba and Bururi | Karuzi, Rutana, Ruyigi, Ngozi, Kirundo | N/A |
| Lesotho | Mokhotlong and Thaba-Tseka | Mafeteng and Mohale's Hoek | Botha-Bothe, Berea, Maseru and Qacha's-nek | N/A |
| Senegal | Kaffrine and Kolda | N/A | Tambacounda, Sédhiou, Kédougou and Ziguinchor | N/A |
| Zambia | Mumbwa, Lufwanyama, Lundazi, Mwense, Mporokoso, Isoka, Mufumbwe, Siavonga, Gwembe and Senanga | N/A | Chadiza, Chavuma, Chibombo, Chinsali, Kazungula, Mpongwe, Mazabuka, Milenge, Mpulungu, and Shangombo | Chilubi, Itezhi-Tezhi, Kalabo, Kapiri Mposhi, Kawambwa, Masaiti, Mwinilunga, Nakonde, Namwala and Nyimba |
| Zimbabwe | Binga, Centenary, Chegutu, Chikomba, Chipinge, Chiredzi, Gokwe South, Gwanda, Gweru Rural, Kariba Nyaminyami, Manangwe, Mazowe, Mutare, Mutoke, Mwenezi and Nkayi | N/A | Bikita, Bindura, Bubi, Chirumhanzu, Hurungwe, Kadoma/Sanyati, Makoni, Matobo, Mt. Darwin, Nyanga, Shurugwi, Umguza, UMP, Umzingwane, Hwedza and Zaka | N/A |

*PBF implementation in Burundi*

Burundi’s PBF program was rolled out in three phases. Bubanza, Cankuzo and Gitega provinces implemented the scheme in December 2006, Makamba and Burui provinces implemented in October 2008, and the remaining provinces implemented in April 2010. This study only includes the first two phases of implementation in order to use the remaining provinces as control areas.

As of 2014, performance-based financing accounts for 40% of the total average health facility budget.^2^ Facilities receive payments based on the quantity and quality of health services provided. Quantity is measured through twenty-three output indicators. Health care facilities report monthly to the Ministry of Health about quantities of health services delivered for each indicator. Reported quantities are verified and validated by a provincial committee through unannounced observation visits to facilities.^2^

In addition to the quantity-based payments, facilities can receive a quality bonus of up to

25 percent.^2^ Quality is assessed quarterly by local regulatory authorities on a randomly chosen day using a checklist containing 220 items grouped into the following topics: general infrastructure and communication, business plan, income and costs, hygiene and sterilization, outpatient consultations, family planning, laboratory services, inpatient care, management of essential drugs, availability of essential drugs, maternal care, surgery, tuberculosis screening,

vaccination, and antenatal care. The total payment to a facility is calculated as a weighted sum

of the number of provided services in the previous three months times their unit payment multiplied by the quality bonus, which ranges between 1 and 1.25 depending on the score obtained from evaluation of facilities based on results of the checklist assessment.^2^

An equity bonus is further used to support facilities that face major problems that cannot be resolved by itself and are critical to its performance.^3^ The calculation of the bonus accounts for geographic remoteness, the poverty of its clients, and the needs of the staff and facility.

Performance bonuses awarded to the staff through the incentive cannot exceed 30% of the health facility’s overall income.^3^

*PBF implementation in Lesotho*

The PBF program in Lesotho had a phased implementation approach. There were two pilot districts; Quthing in April 2014 and Leribe in January 2015. These two districts are excluded from this study. Mokhotlong and Thaba-Tseka began implementation in July 2016 and Mafeteng and Mohale’s Hoek began implementation in October 2016, which are analyzed in this study.

PBF payments are determined based on the quantity of services provided, quality of services provided, and for health centers, the relative remoteness of the health center.^4^ Quantity performance is assessed monthly, quality performance is measured quarterly, and payments are made quarterly. Facilities report fourteen quantity indicators, which are then verified by the Performance Purchasing Technical Assistance team. There are per-unit incentives for each service that are adapted based on facility performance so that, there are higher incentives for lower performing indicators and small incentives when the service is near capacity.

The quality score is based on a quality assessment checklist (80% weight) and a client satisfaction survey (20% weight).^4^ The assessment checklist and satisfaction tools are specific to the level of the health system (health center vs hospital), and the checklists are reviewed every year for changes to the indicators or revising the points assigned to indicators. The quality performance score translates to a graduated bonus on top of the quantity incentive payment. Health centers receive no quality bonus for quality scores below 50, and 65% bonus for quality scores between 90-100. Hospitals receive no bonus for quality scores below 50 and the full bonus for quality scores between 95-100.

In addition, health centers in remote areas receive additional bonuses to address inequities in retaining staff, higher transportation and communication costs and limited access to other services.^4^ Health centers located outside urban areas but with access to public transport and network receive a 10% bonus over the quantity produced (not quality), while those in remote areas with infrequent public transportation and unreliable network receive a 20% bonus. Health centers in urban areas and hospitals receive no remoteness bonuses.

A minimum of 50% of the incentive payments must be used for improvement of service delivery, and a maximum of 50% may be used for motivation bonuses for health center or hospital staff. In addition, district health management teams may receive PBF incentives with an emphasis on quality of supportive supervision and essential support for the PBF project.

*PBF implementation in Senegal*

Senegal’s PBF program was piloted in two regions, Kaffrine and Kolda, in April 2012, and was expanded to four additional regions in May 2016.^5^ Only the two pilot regions were evaluated in this study.

PBF payments are based on both coverage and quality targets. Coverage targets are set for each health facility based on the previous year’s target. Payments are disbursed if quarterly and annual coverage targets are met, and are deflated by a quality score. Quantity and quality performance is reported by the facility and verified by the Regional Management Committee, facility visits, and household surveys. 25% of PBF payments must be used to cover operational costs, and a maximum of 75% can be used to pay individual incentives to health workers.^6^

In addition to the supply-side incentives, Senegal’s program also incorporates demand-side incentives, with vouchers for four antenatal care visits and skilled deliveries.^5^

*PBF implementation in Zambia*

Zambia’s PBF evaluation contained three arms: an PBF intervention group that received PBF performance-based grans and Emergency Obstetric and Neonatal Care (EmONC) equipment; an unconditional financing arm that received the EmONC equipment and funding equivalent to the average of the PBF performance grants as input financing; and a pure control group that received nothing.^7^ This study uses the pure control as the comparison districts in the main analysis and the unconditional financing group in a secondary analysis.

Ten districts were randomized to each evaluation arm. Districts selected for the evaluation approximated the median population health, socio-economic condition, and health governance capacity for the provinces in which they were located. Three districts in each rural province were selected as well as six districts in Northern and Southern provinces; within each province the selected districts were then randomized to either the PBF intervention or one of the two treatment arms.

The PBF program used a contracting-in strategy.^7^ PBF payments were based on nine maternal and child health output indicators and ten dimensions of quality. Quantity unit incentives ranged from USD 0.20 for curative consultations to USD 6.40 for institutional deliveries by skilled birth attendants. The ten quality indicators were assigned different weights, then received bonuses on top of the quantity payments, with a quality score of 61%-69% corresponding to an additional 15%, scores of 70%-79% received an additional 25% and scores over 80% received an additional 50% on top of the quantity payments. A small number of more remote health facilities were randomly assigned to receive 25% higher prices for all output indicators. In addition, the district medical offices received performance bonuses for fulfilling a set of supervision and management functions. Quantity and quality scores were externally verified.

Health facilities were required to spend a minimum of 40% of their PBF payments on operational activities, while a maximum of 60% could be spent on staff motivational bonuses.^7^

*PBF implementation in Zimbabwe*

After PBF pilots in two districts, Zimbabwe’s PBF program was implemented in 16 districts. The pilot districts were excluded from this study.^8^ 32 districts were purposively selected from the universe of 64 districts and pair-matched on the following characteristics: geographic accessibility, type and level of health facilities, average facility catchment population, proportion of staff in position, presence of key staff such as the district medical/health officer, and health service utilization rates for antenatal and postnatal care coverage, institutional delivery and immunization rates. One district in each pair was selected by the Ministry of Health and Child Care into the PBF treatment arm, while the others were used as control districts.

PBF payments were based on quantity, quality and a remoteness bonus.^8^ Rural health centers received a unit price for 16 quantity indicators, while district hospitals quantity payments were based on five indicators mostly related to deliveries. Remoteness bonuses up to 30% of the quantity payments were based on population density, distance to the nearest referral facility and availability of roads, public transportation and communications. Quality of services was measured through a client satisfaction survey and a balanced score card covering structural quality, process quality, organization and management systems. Quality scores translated into bonuses on top of the quantity and remoteness payments of up to 25%.

Health facilities were required to spend a minimum of 75% of their PBF payments on improving working conditions at the facility, while up to 25% could be spent on staff bonuses.^8^

In addition to the PBF payments, intervention districts received training on effective PBF implementation and abolishment of formal user fees for services that PBF was targeting.^8^

**Appendix 2. Trends in secondary outcomes over study period**

Facility delivery trends


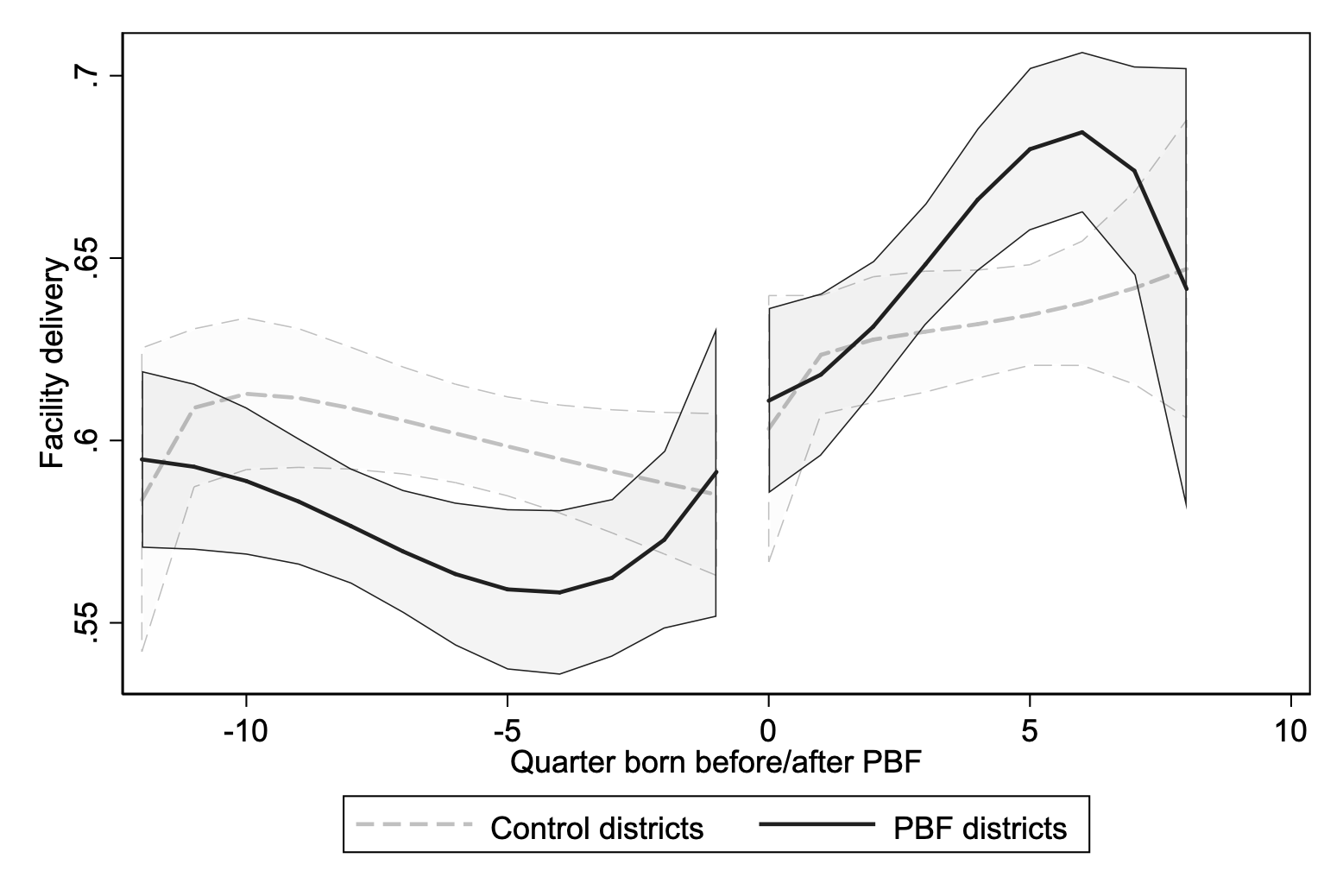


Delivery quality trends


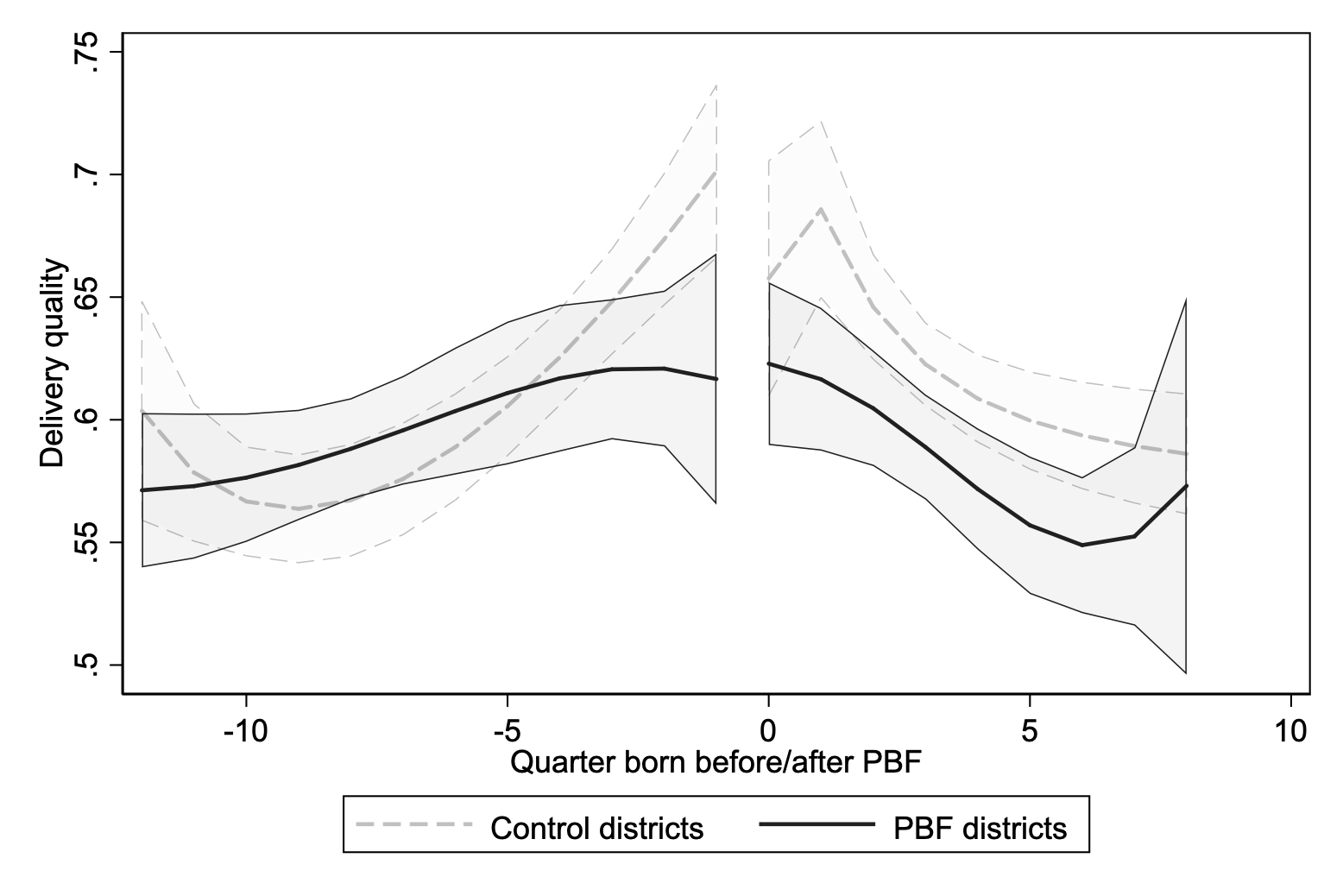


Cesarean section trends


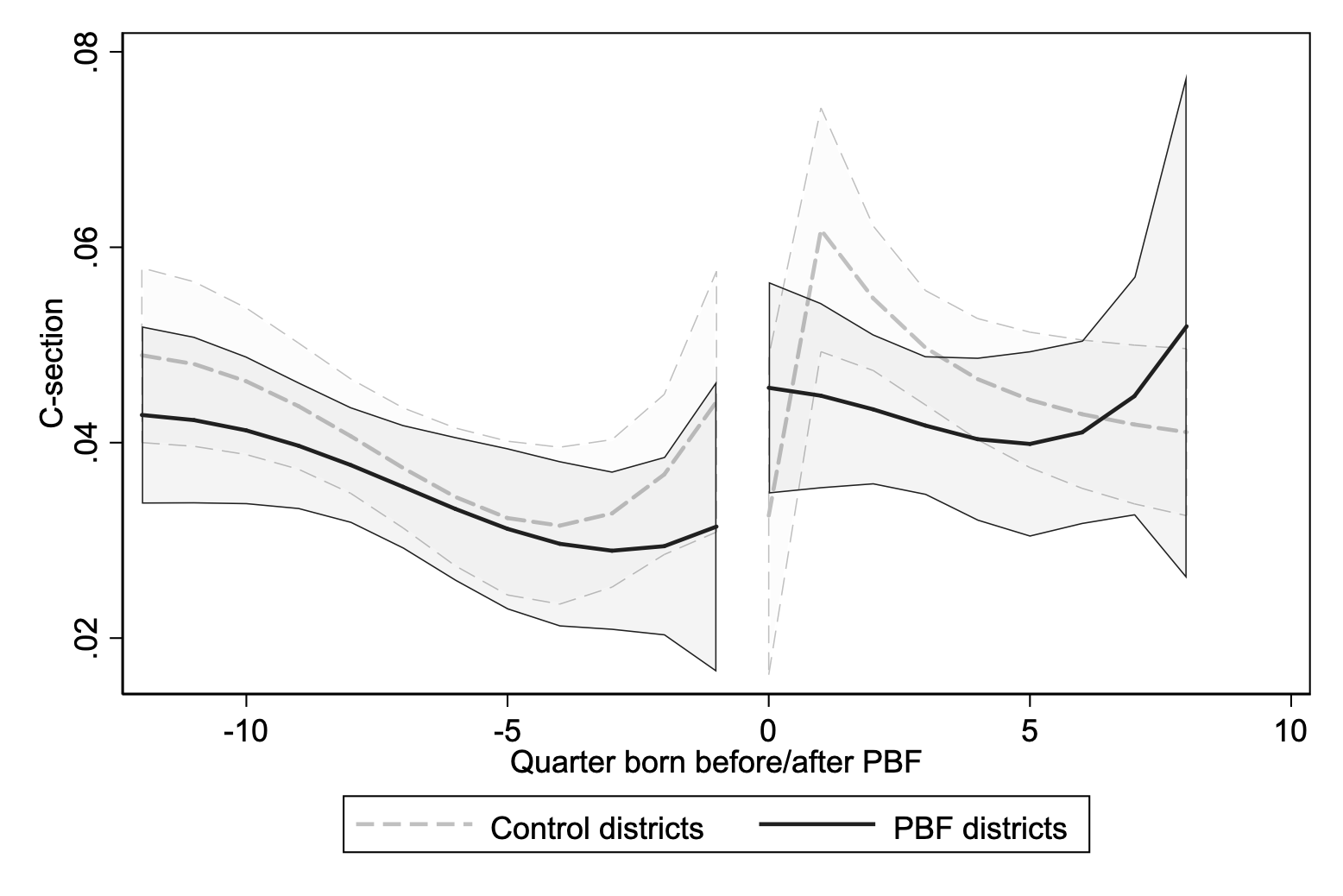


Antenatal care utilization trends


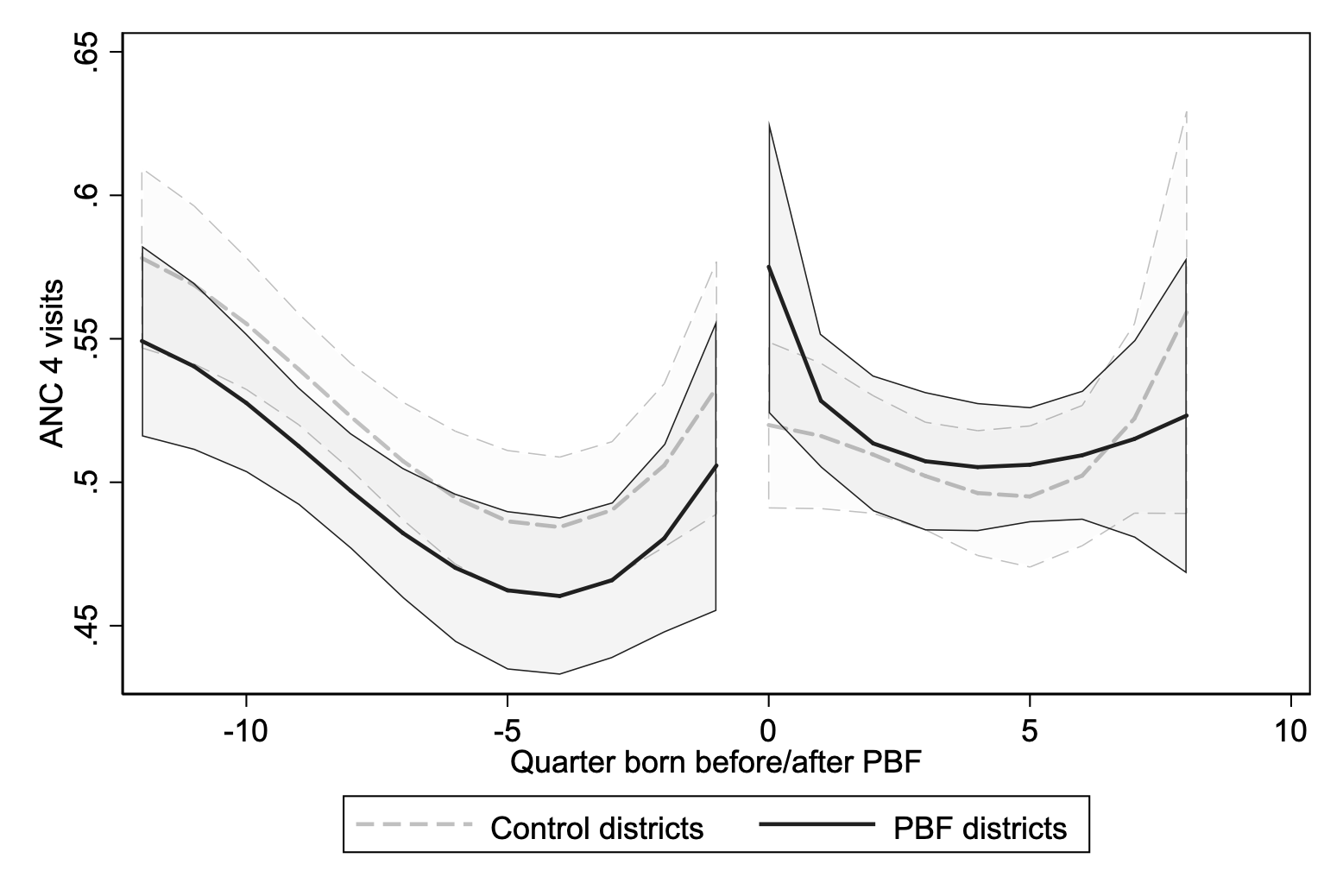


Antenatal care quality trends


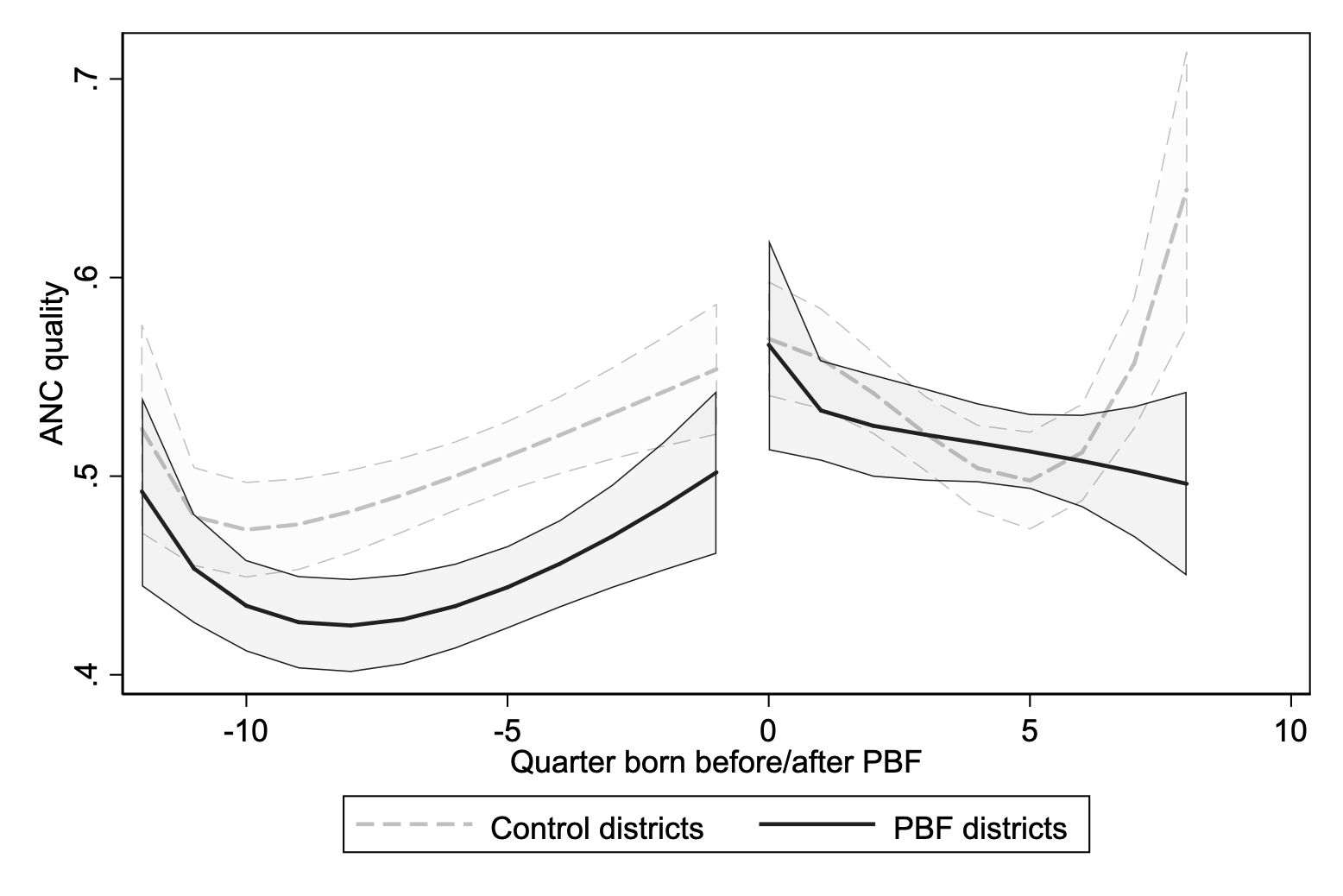


**Appendix 3. Modeled effects in pre-trends and over time**

These graphs show the estimated effect of PBF relative to time zero (start of PBF implementation), using a multi-period difference and difference framework. Dummy indicators are included in the models for PBF implementation interacted with the quarter of interest, along with month and district fixed effects. These can be used to evaluate if there were differences in trends prior to implementation (blue points and confidence intervals), or if there were any delayed PBF effects (red).

**Appendix 4. Robustness checks**

Pooled impact on alternative quality measures and birthweight measurement

| Outcome | Percentage point change | 95% CI | N |
| --- | --- | --- | --- |
| Low birthweight (recorded observations only) | 0.012 | (-0.02,0.04) | 11226 |
| Delivery quality (mean)^a^ | -0.019 | (-0.05,0.01) | 13054 |
| ANC quality (mean)^a^ | 0.015 | (-0.01,0.04) | 14510 |
| Birthweight recorded^b^ | 0.011 | (-0.1,0.12) | 28619 |

^a^Defined as the percent of delivery or ANC items received

^b^Defined as whether birthweight copied from a record (as opposed to mother’s report)

Pooled results in all study countries except for Burundi

| Outcome | Percentage point change | 95% CI | N |
| --- | --- | --- | --- |
| Early neonatal death | 0.00 | (-0.01,0.01) | 20331 |
| Low birthweight | 0.01 | (-0.01,0.03) | 20331 |
| Facility delivery | 0.03 | (-0.01,0.07) | 19331 |
| Delivery quality | -0.05 | (-0.14,0.05) | 11835 |
| C-section | 0.00 | (-0.02,0.01) | 19279 |
| ANC 4 visits | 0.04 | (-0.02,0.10) | 13590 |
| ANC quality | 0.02 | (-0.05,0.09) | 13714 |

Pooled results using all non-implementation districts in the country as a comparison

| Outcome | Percentage point change | 95% CI | N |
| --- | --- | --- | --- |
| Early neonatal death | 0.00 | (-0.00,0.01) | 64194 |
| Low birthweight | 0.00 | (-0.01,0.02) | 64194 |
| Facility delivery | 0.04 | (-0.00,0.07) | 51730 |
| Delivery quality | -0.03 | (-0.10,0.04) | 35446 |
| C-section | 0.00 | (-0.01,0.01) | 51478 |
| ANC 4 visits | 0.04 | (-0.00,0.08) | 34491 |
| ANC quality | 0.03 | (-0.01,0.08) | 34902 |

Pooled results using second implementation date in Burundi and Lesotho

| Country | Outcome | Percentage point change | 95% CI | N |
| --- | --- | --- | --- | --- |
| Burundi | Early neonatal death | 0.00 | (-0.01,0.01) | 5588 |
| Burundi | Low birthweight | 0.01 | (-0.12,0.13) | 5588 |
| Burundi | Facility delivery | -0.03 | (-0.1,0.04) | 2154 |
| Burundi | Delivery quality | 0.02 | (-0.16,0.19) | 1212 |
| Burundi | C-section | -0.01 | (-0.02,0.01) | 2160 |
| Burundi | ANC 4 visits | 0.10 | (-0.01,0.21) | 1263 |
| Burundi | ANC quality | 0.12 | (0.07,0.18) | 1267 |
| Lesotho | Early neonatal death | 0.00 | (-0.04,0.04) | 1899 |
| Lesotho | Low birthweight | -0.10 | (-0.19,-0.01) | 1899 |
| Lesotho | Facility delivery | 0.06 | (-0.01,0.13) | 1213 |
| Lesotho | Delivery quality | -0.16 | (-0.27,-0.05) | 1054 |
| Lesotho | C-section | -0.03 | (-0.21,0.15) | 1213 |
| Lesotho | ANC 4 visits | 0.13 | (-0.07,0.34) | 1213 |
| Lesotho | ANC quality | -0.08 | (-0.39,0.22) | 1230 |

PBF vs unconditional financing in Zambia

| Outcome | Percentage point change | 95% CI | N |
| --- | --- | --- | --- |
| Early neonatal death | 0.00 | (-0.03,0.02) | 3432 |
| Low birthweight | -0.02 | (-0.09,0.05) | 3432 |
| Facility delivery | 0.05 | (-0.05,0.14) | 3418 |
| Delivery quality | -0.06 | (-0.15,0.03) | 2282 |
| C-section | -0.01 | (-0.04,0.01) | 3424 |
| ANC 4 visits | 0.01 | (-0.09,0.11) | 2267 |
| ANC quality | 0.06 | (-0.02,0.14) | 2307 |

**Appendix 5.** Ex-post power calculation assumptions

We conducted ex-post power calculations to estimate the minimum detectable effect (MDE) possible with the available sample size. We calculated the MDE with the following assumptions:

1. The observations are clustered based on the actual number of districts in the study: 40 treatment and 35 control
2. The sample size is based on the actual number of matched observations for each dependent variable, see table
3. Initial mean and rho are calculated from all matched observations at baseline, see table
4. Power=0.80, Alpha=0.05

Minimum detectable difference and assumptions

|  | N treat | N control | Mean | Rho | MDE |
| --- | --- | --- | --- | --- | --- |
| Early neonatal death | 13164 | 18484 | 0.022 | 0.003 | -0.007 |
| Low birthweight | 7853 | 11842 | 0.182 | 0.003 | -0.020 |
| Facility delivery | 10074 | 12607 | 0.586 | 0.037 | 0.064 |
| Delivery quality | 6167 | 7663 | 0.601 | 0.046 | 0.073 |
| C-section | 10040 | 12595 | 0.036 | 0.009 | 0.012 |
| ANC 4 visits | 6948 | 8585 | 0.512 | 0.017 | 0.048 |
| ANC quality | 6999 | 8664 | 0.481 | 0.020 | 0.051 |

**References**

1 Josephson E, Gergen J, Coe M, Ski S, Madhavan S, Bauhoff S. How do performance-based financing programmes measure quality of care? A descriptive analysis of 68 quality checklists from 28 low- and middle-income countries. *Health Policy Plan* 2017; **32**: 1120–1126.

2 Bonfrer I, Van de Poel E, Van Doorslaer E. The effects of performance incentives on the utilization and quality of maternal and child care in Burundi. *Soc Sci Med* 2014; **123**: 96–104.

3 Manuel des Procedures Pour la Mise en Oeuvre du Financement Base sur la Performance Seconde Generation. Ministere de la Sante Publique et de la Lutte Contre le Sida, Republique du Burundi, 2017 http://www.fbpsanteburundi.bi/cside/contents/docs/Manuel_des_procedures_FBP_seconde_generation.pdf (accessed Nov 3, 2020).

4 Lesotho Ministry of Health. Maternal and Newborn Health Performance Based Financing Project User Guide Manual. 2016 https://www.rbfhealth.org/sites/rbf/files/documents/Lesotho-Maternal-Newborn-Health-PBF-Project-User-Manual.pdf.

5 RBF Health. Senegal. 2017. https://www.rbfhealth.org/rbfhealth/country/senegal.

6 El-Khoury M, Faye S, Baruwa E. Results-Based Financing, Senegal: A look inside the ‘black box’. Rabat, Morocco, 2016. https://afhea.org/docs/presetationspdfs/Sophie%20-%20Results-Based%20Financing,%20Senegal-%20%20A%20look%20inside%20the%20%E2%80%98black%20box%E2%80%99.pdf.

7 Friedman J, Qamruddin J, Chansa C, Das AK. Impact evaluation of Zambia’s health results-based financing pilot project. *Washington, DC: World Bank Group* 2016.

8 Friedman J, Das A, Mutasa R. Rewarding Provider Performance to Improve Quality and Coverage of Maternal and Child Health Outcomes: Zimbabwe Results-Based Financing Pilot Program. The World Bank, 2016.
